# Structure of the tandem PX-PH domains of Bem3 from *Saccharomyces cerevisiae*

**DOI:** 10.1107/S2053230X18005915

**Published:** 2018-04-24

**Authors:** Imtiaz Ali, Sungmin Eu, Daniel Koch, Nathalie Bleimling, Roger S. Goody, Matthias P. Müller

**Affiliations:** a Department of Structural Biochemistry, Max Planck Institute of Molecular Physiology, Otto-Hahn-Strasse 11, 44227 Dortmund, Germany; b Faculty of Chemistry and Chemical Biology, TU Dortmund University, Otto-Hahn-Strasse 4a, 44227 Dortmund, Germany

**Keywords:** PX domain, PH domain, phox, pleckstrin homology, phosphatidylinositol phosphates, PIP, *Saccharomyces cerevisiae*, Bem3

## Abstract

The structure of the tandem lipid-binding PX and pleckstrin-homology (PH) domains of the Cdc42 GTPase-activating protein Bem3 from *Saccharomyces cerevisiae* (strain S288c) has been determined to a resolution of 2.2 Å (*R*_work_ = 21.1%, *R*_free_ = 23.4%). It shows that the domains adopt a relative orientation that enables them to simultaneously bind to a membrane and suggests possible cooperativity in membrane binding.

## Abstract 2

The structure of the putative membrane-binding tandem PX-PH domain module of the yeast protein Bem3 is reported.

## 1. Introduction   

**Figure 1:**
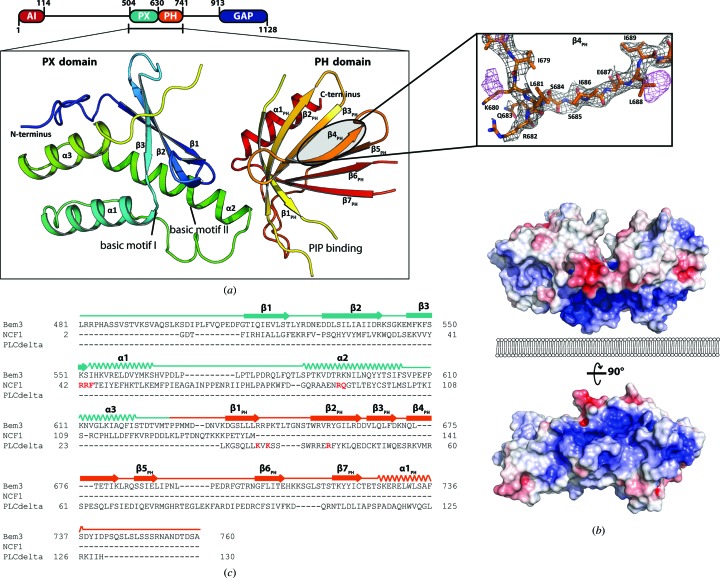
(*a*) Domain architecture of Bem3 and cartoon representation of the PX and PH domains of Bem3 and their relative positions within the full-length protein. Important regions that are putatively involved in membrane binding are indicated for both domains. The inset additionally shows a magnified view of β4_PH_ where the electron density did not allow unambiguous assignment of the amino-acid side chains (2*mF*_o_ − *DF*_c_ electron density shown in black at an r.m.s.d. of 1). Therefore, the anomalous signal from the SeMet-derivative crystals was used to correctly assign the position of amino acids (anomalous map in magenta depicted at an r.m.s.d. of 4). (*b*) Electrostatic potential of Bem3 contoured at ±5*kT* e^−1^ calculated with *APBS* (Baker *et al.*, 2001 ▸) shown in two orientations including the highly basic putative membrane-binding site [the upper figure is shown in the same orientation as in (*a*), the membrane is indicated below the structure]. All structural representations were prepared with *PyMOL* (DeLano & Lam, 2005 ▸). (*c*) Sequence alignment produced with *PROMALS*3*D* (Pei *et al.*, 2008 ▸) of Bem3 with the PX domain of Ncf1 (UniProt ID P14598) and the PH domain of PLCδ (UniProt ID P10688). Important residues that belong to the basic motifs I and II (PX domain; Sato *et al.*, 2001 ▸) and the PIP-binding region within the PH domain (Lemmon, 2007 ▸) are highlighted in red.

The Bem3 protein from *Saccharomyces cerevisiae* (UniProt ID P32873) is a large multi-domain protein consisting of 1128 amino acids (Zheng *et al.*, 1994 ▸). Three domains within the protein can be deduced from the amino-acid sequence: a PX domain (amino acids 504–630), a PH domain (amino acids 630–741) and a RhoGAP domain at the C-terminus of Bem3 (amino acids 913–1128) (Fig. 1 ▸). It has also been suggested that the N-terminal part of the protein (amino acids 1–114) is an auto-inhibitory module which results in a constitutively active variant of Bem3 upon deletion (Fig. 1 ▸; Kadota *et al.*, 2004 ▸; Mukherjee *et al.*, 2013 ▸).

The PX domain, which was discovered as a conserved sequence of 100–140 amino acids (Ponting, 1996 ▸), has been identified in more than 100 yeast and mammalian proteins belonging to diverse families (Ellson *et al.*, 2002 ▸; Sato *et al.*, 2001 ▸; Seet & Hong, 2006 ▸; Xu *et al.*, 2001 ▸). The conserved three-dimensional structure contains an N-terminal β-sheet followed by a helical subdomain with three α-helices (Lu *et al.*, 2002 ▸). Conserved basic residues present in the β3–α1 region of the PX domain are believed to mediate interaction with the phosphate groups of phosphatidylinositol phosphates (PIPs) within membranes (Cheever *et al.*, 2001 ▸; Ellson *et al.*, 2001 ▸; Song *et al.*, 2001 ▸; Xu *et al.*, 2001 ▸). These PIPs are phosphoryl­ated derivatives of phosphatidylinositol that constitute a small fraction of the total cellular lipids but are important components of cellular organization (Lenoir & Overduin, 2013 ▸). Phosphorylation at the 3′-, 4′- and 5′-hydroxyl groups of the inositol ring leads to seven different phosphatidylinositol phosphates. Each of these singly, doubly or triply phosphorylated PIPs displays a distinct localization in cellular organelles, where they recruit specific PIP-binding effectors to exert their regulatory functions, such as lipid signalling, vesicle transport and cell growth (Lenoir & Overduin, 2013 ▸; Mayinger, 2012 ▸). The specificity and affinity towards certain PIPs can vary between different PX domains (Song *et al.*, 2001 ▸). Whereas many PX domains exhibit a common preference for PI3P as the primary binding partner on membranes, others bind less specifically and recognize different PIPs (Mayinger, 2012 ▸). The low binding affinity for PI3P observed in some PX-domain-containing proteins is associated with the presence of a secondary lipid-binding site within this domain which specifically binds to phosphatidic acids (PAs) or phosphatidylserine (PS), as well as other PIPs, and can synergistically cooperate with the primary PI3P binding of the PX domain (Karathanassis *et al.*, 2002 ▸; Stahelin *et al.*, 2004 ▸). Apart from the secondary lipid-binding site, the possible oligomerization of some proteins owing to the PX domain can further enhance the interaction between these proteins and membranes (Xing *et al.*, 2004 ▸).

The second important PIP-binding domain found in Bem3 is the pleckstrin-homology (PH) domain, which directly follows the PX domain within the primary sequence of Bem3 and consists of approximately 100 amino acids (Haslam *et al.*, 1993 ▸). The structure of PH domains consists of seven strongly bent antiparallel β-strands, which are terminated by a C-terminal α-helix (Blomberg *et al.*, 1999 ▸; Macias *et al.*, 1994 ▸; Saraste & Hyvönen, 1995 ▸; Yoon *et al.*, 1994 ▸). Sequence-similarity searches in the human protein database led to the identification of this domain in more than 250 proteins, making it the 11th most abundant domain in the human proteome (Letunic *et al.*, 2006 ▸). PH domains preferably bind to PIPs that have adjacent phosphates, such as PI(4,5)P_2_, PI(3,4)P_2_ and PI(3,4,5)P_3_. However, only a minor fraction of these domains show high affinity and specificity for one PIP, whereas other PH domains either do not recognize PIPs or interact very weakly and the functional significance of their interactions is not clear (Isakoff *et al.*, 1998 ▸; Kavran *et al.*, 1998 ▸; Lemmon & Ferguson, 2000 ▸; Takeuchi *et al.*, 1997 ▸; Yu *et al.*, 2004 ▸). In addition to PIP binding, the β-sandwich structure of PH domains is involved in various other functions, including protein–protein interactions as well as oligomerization (Baumeister *et al.*, 2003 ▸; Klein *et al.*, 1998 ▸; Worthylake *et al.*, 2004 ▸, Lemmon, 2007 ▸).

In this study, we have determined the X-ray crystallographic structure of the interesting tandem PX-PH domain module of Bem3 to a resolution of 2.2 Å, showing that the putative membrane-binding regions of both domains are oriented in the same direction and thus might act synergistically in membrane recognition and binding.

## 2. Experimental procedures   

### 2.1. Plasmid construction   

Bem3 constructs encompassing the regions containing amino acids 500–765 or 500–750 (comprising the PX and PH domains of Bem3) were amplified from yeast genomic DNA (strain S288c) using *Pfu* polymerase (NEB, England) and the primers listed in Table 1 ▸. The amplicons were digested with the respective restriction enzymes (NdeI, BamHI and XhoI; the recognition sequences are underlined in the primer sequences) and incorporated into a modified bacterial expression plasmid (pET-19mod). The resulting constructs were used for the expression of Bem3 including an N-terminal hexahistidine tag followed by a *Tobacco etch virus* (TEV) protease cleavage site. For phasing and unambiguous assignment of the amino-acid sequence in the structure, point mutations were introduced by site-directed mutagenesis at positions 680 (K680M) and 688 (L688M) to incorporate l-selenomethionine (SeMet).

### 2.2. Protein purification   

To express the recombinant proteins, *Escherichia coli* BL21-CodonPlus (DE3)-RIL cells were transformed with the corresponding plasmid and allowed to grow at 37°C until the OD_600_ reached approximately 0.60–0.65. Expression was induced by adding isopropyl β-d-1-thiogalactopyranoside (IPTG) to a final concentration of 0.2 m*M* and growth was continued overnight at 20°C. The SeMet-labelled mutants were expressed using conditions described elsewhere (Van Duyne *et al.*, 1993 ▸).

The cells were harvested by centrifugation, resuspended in cell-lysis buffer [50 m*M* HEPES pH 8, 500 m*M* LiCl, 5%(*v*/*v*) glycerol, 2 m*M* β-mercaptoethanol (β-ME), 1 m*M* PMSF and a protease-inhibitor cocktail including 50 µg ml^−1^ chymo­statin, leupeptin, antipain and pepstatin (Sigma)] and disrupted in a fluidizer (Microfluidics). The lysate was incubated with 1% CHAPS for 30 min. Insoluble material was removed by centrifugation at 50 000*g* for 50 min at 10°C and the supernatant was applied onto an Ni^2+^–NTA HisTrap column pre-equilibrated with buffer *A* [50 m*M* HEPES pH 8, 500 m*M* LiCl, 5%(*v*/*v*) glycerol, 2 m*M* β-ME]. After extensive washing of the column, His-tagged proteins were eluted with a linear gradient of buffer *B* (buffer *A* supplemented with 500 m*M* imidazole). The polyhistidine tag was cleaved overnight at 4°C using 0.06 mg TEV protease per milligram of recombinant protein during dialysis [the dialysis buffer consisted of 50 m*M* HEPES pH 8, 100 m*M* NaCl, 5%(*v*/*v*) glycerol, 2 m*M* β-ME]. After a second Ni^2+^–NTA affinity purification to remove uncleaved protein and TEV protease, final purification was achieved by size-exclusion chromatography (Superdex 200 16/60, GE Healthcare) in the final storage buffer [20 m*M* HEPES pH 8, 100 m*M* NaCl, 5%(*v*/*v*) glycerol, 2 m*M* dithioerythritol].

### 2.3. Crystallization   

To determine the initial crystallization conditions, Bem3 was subjected to crystallization trials at 293 K by the sitting-drop vapour-diffusion method using the following commercially available protein crystallization screens: The PACT Suite, The PEGs Suite and The JCSG Core I–IV Suites (Qiagen, Hilden, Germany). The crystallization drops were prepared by mixing 100 nl protein solution with 100 nl reservoir solution with the help of a TTP Labtech Mosquito LCP crystal liquid-handling robot and were incubated against 70 µl mother liquor in 96-well plates. The best condition [0.1 *M* PCB (sodium propionate, sodium cacodylate and bis-tris propane in a 2:1:2 molar ratio; Qiagen) pH 8.0, 25% PEG 1500] obtained from the initial screen was further improved by varying the pH and the concentration of PEG. The best diffracting crystals of wild-type Bem3 and the SeMet derivative grew in the form of hexagonal rods within 1 d. The final crystallization conditions are summarized in Table 2 ▸.

### 2.4. Data collection and processing   

Individual hexagonal rods were harvested, cryoprotected in mother liquor supplemented with 10%(*v*/*v*) glycerol and flash-cooled in liquid nitrogen prior to data collection. The Bem3 crystals were maintained at 100 K and the collection of X-ray diffraction data was performed on beamline X10SA at the Swiss Light Source (SLS), Paul Scherrer Institute, Villigen, Switzerland. The data were indexed and integrated with *XDS* and scaled with *XSCALE* (Kabsch, 2010*a* ▸,*b* ▸). Data statistics are described in Table 3 ▸.

### 2.5. Structure solution and refinement   

Initial phases and an initial model were obtained using the data set from the SeMet derivative of L688M Bem3_500–750_ (containing a total of six SeMet residues) with *phenix.autosol* (Terwilliger *et al.*, 2009 ▸). This resulted in an initial model with 82 amino-acid residues built (BAYES-CC = 44.3 ± 9.6, *R*_work_ = 34.3%, *R*_free_ = 37.9%) and was followed by automated model building in *phenix.autobuild* (*R*_work_ = 27.2%, *R*_free_ = 30.0%) (Terwilliger *et al.*, 2008 ▸). The model was iteratively improved by manual model building in *Coot* (Emsley *et al.*, 2010 ▸) and refinement with *phenix.refine* (Afonine *et al.*, 2005 ▸) against the native data set for Bem3_500–765_. Validation of the structure was performed with *MolProbity* (Chen *et al.*, 2010 ▸). Refinement statistics can be found in Table 4 ▸.

## 3. Results and discussion   

The crystal structure of the tandem PX-PH domains shows the well characterized folds of both protein domains as reported previously (Riddihough, 1994 ▸; Bravo *et al.*, 2001 ▸). The model contains amino acids 502–629 belonging to the PX domain and 636–737 of the PH domain, but the linker region (amino acids 630–635) between the domains as well as the loops between β1_PH_ and β2_PH_ (amino acids 647–651) and β6_PH_ and β7_PH_ (amino acids 709–715) within the PH domain were flexible and could not be resolved. Even though six amino acids are missing between the two domains, the presented structural assembly within the asymmetric unit represents the only possible arrangement, and a different connectivity between the PX and PH domains is excluded because the positions of the respective C- and N-termini are too remote. Using the native data set only, the sequence could not be unambiguously assigned within parts of the PH domain because of a lack of electron density for the side chains (especially within β4_PH_; see Fig. 1 ▸). We therefore used the anomalous signal from crystals of SeMet-labelled K680M Bem3_500–750_ and L688M Bem3_500–750_ in order to correctly assign the positions of amino acids in this region (the electron density is shown in Fig. 1 ▸ *a*).

As outlined in §[Sec sec1]1, both the PX and PH domains have been implicated in binding to PIPs and, besides other possible functions, mediate membrane binding and localization in cells. Strikingly, the PX domain of Bem3 contains basic residues (for example Lys551 and Arg592/Lys593) at similar positions compared with other PX domains, which have previously been termed basic motifs I and II, respectively, and have been shown to be involved in PIP binding (Sato *et al.*, 2001 ▸). A similar observation also holds true for the PH domain (basic residues Arg644, Arg645 and Arg658), with similar relative positions compared with the PH domain of, for example, PLCδ (see the alignment in Fig. 1 ▸ *c*; Lemmon, 2007 ▸). Positively charged residues within this part of the protein consisting of strands β1_PH_ and β2_PH_ and the connecting loop have also previously been identified as an important lipid-binding motif (Isakoff *et al.*, 1998 ▸). This motif consists of the first lysine at the end of β1_PH_ and an arginine in the middle of β2_PH_, which both interact with adjacent phosphate head groups of PIP2 (Ferguson *et al.*, 1995 ▸, 2000 ▸). The canonical lysine is, however, replaced by an arginine in the PH domain of Bem3. This arginine (Arg644) is well conserved among PH domains lacking the canonical lipid-binding sequence (Anand *et al.*, 2012 ▸; Macia *et al.*, 2008 ▸). The crystal structure of the PH domain of Slm1 (PDB entry 4a6k) revealed that the corresponding Arg478 interacts with PIPs (Anand *et al.*, 2012 ▸). Specific point mutations introduced at this position in Slm1 (Gallego *et al.*, 2010 ▸) and the PH domain of EFA6 (Macia *et al.*, 2008 ▸) resulted in mislocalization of the respective proteins, further signifying the role of this arginine in ligand binding. In line with these findings, an analysis involving the mutation of basic residues within the β1_PH_–β2_PH_ loop of yeast Bem3 (R644S, R645S and K647D) showed that this abolished binding of the PH domain to PIP2, rendering the protein incapable of correctly localizing to the polar site at the cell membrane (Mukherjee *et al.*, 2013 ▸). In contrast to these mutations within the PH domain, a quadruple mutant (Y524W, R578S, L580S and F581M; Mukherjee *et al.*, 2013 ▸) involving amino acids within the so-called ‘membrane-interaction loop’ (Cheever *et al.*, 2001 ▸) of the PX domain exhibits wild-type localization of Bem3. Although the critical residues Lys551 and Arg592 of basic motifs I and II of the Bem3 PX domain are conserved in the majority of PX domains (Cheever *et al.*, 2001 ▸), their role in the direct binding of PIPs has yet to be elucidated.

**Figure 2:**
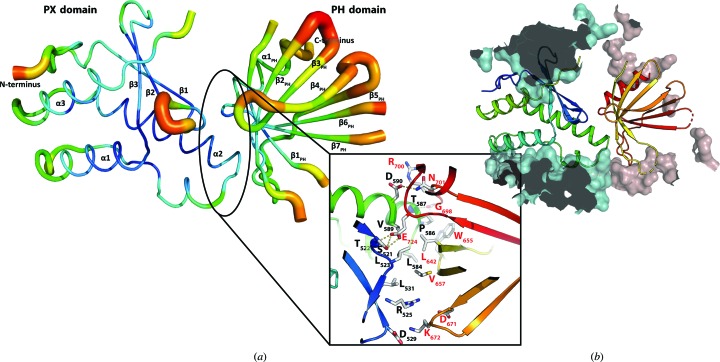
(*a*) *B*-factor representation of the PX and PH domains of Bem3 [putty representation from *PyMOL*; colours indicate low *B* factors (blue) to high *B* factors (red)]. The average *B* factor for the whole structure is 81 Å^2^ (PX domain, 71 Å^2^; PH domain, 96 Å^2^). Additionally, amino acids within the interface between the domains are shown. (*b*) Surfaces of neighbouring molecules in the crystal show that the PX domain has large contact areas (cyan surfaces), whereas the PH domain does not (red surfaces).

Interestingly, the putative membrane-binding sites of both domains (basic motifs I and II within the PX domain and the PIP-binding site within the PH domain) are oriented in a similar direction (Fig. 1 ▸ *a*) and this part of the protein displays a large positively charged basic surface (Fig. 2 ▸ *b*). The similar orientation thus suggests a possible synergy between the domains with regard to the recognition of PIPs within a membrane, both with respect to the affinity of binding and the possibility of the recognition of a distinct membrane or compartment within the cell by the relative abundance of PIPs.

The interface of ∼620 Å^2^ between the PX and PH domains (calculated using *PDBePISA*; Krissinel & Henrick, 2007 ▸) contains several hydrophobic interactions between Leu523, Leu531, Leu584, Pro586 and Val589 in the PX domain, and Leu642, Trp655 and Val657 in the PH domain, as well as hydrogen bonds (Thr587–Gly698, Ser521–Glu724, Thr522–Glu724 and Asp590–Asn701) and ionic interactions (Arg525–Asp671, Asp529–Lys672 and Asp590–Arg700) (Fig. 2 ▸), and many of these amino acids are conserved among Bem3 proteins from different organisms. Interestingly, a flexible relative mobility of the domains is indicated by the elevated *B* factors, especially at the periphery of the PH domain facing away from the PX domain (Fig. 2 ▸ *a*), resulting in average *B* factors of 71 and 96 Å^2^ for the PX and PH domains, respectively. The elevated *B* factors of the PH domain can be understood by considering the different crystal contacts. Whereas the PX domain has large contact areas (∼1210 Å^2^) with symmetry-related molecules, the PH domain has only small contact areas (∼310 Å^2^; contact areas were calculated with *PDBePISA*; Krissinel & Henrick, 2007 ▸; Fig. 2 ▸ *b*), thus allowing the increased mobility. While we can only speculate on the possible role of this flexibility, it is conceivable that a less-than-rigid relative orientation of the two lipid-binding domains might allow different membrane curvatures in different specific situations.

## 4. Conclusions   

In this publication, we present the three-dimensional structure of the tandem PX-PH domains of the yeast protein Bem3. The structure shows that the putative lipid-binding domains adopt a relative orientation that enables them to simultaneously bind to a membrane. Therefore, future studies should address the possibility of cooperativity between the domains with regard to membrane interaction as well as the resulting specificity with respect to the relative abundances of PIPs in a target membrane.

## Supplementary Material

PDB reference: tandem PX-PH domains of Bem3 from *S. cerevisiae*, 6fsf

## Figures and Tables

**Table 1:** Macromolecule-production information Recognition sequences are underlined in the primer sequences.

	Bem3_500–765_	Bem3_500–750_
DNA source	Genomic DNA (strain S288c)	Genomic DNA (strain S288c)
Forward primer	AAAAAACATATGAAGTCGGATATTCCATTATTCGTTCAACCGG	AAAAAACATATGAAGTCGGATATTCCATTATTCGTTCAACCGG
Reverse primer	AAAAAAGGATCCTCATGCGCTCAAGTGAGATGCAGAATCAGTATCG	AAAAAACTCGAGTCAGCTAGATAACGATAAACTTTGTGAAGGATCAATG
Expression vector	pET-19mod	pET-19mod
Expression host	*E. coli* BL21-CodonPlus (DE3)-RIL	*E. coli* BL21-CodonPlus (DE3)–RIL
Complete amino-acid sequence of the construct produced	GHMKSDIPLFVQPEDFGTIQIEVLSTLYRDNEDDLSILIAIIDRKSGKEMFKFSKSIHKVRELDVYMKSHVPDLPLPTLPDRQLFQTLSPTKVDTRKNILNQYYTSIFSVPEFPKNVGLKIAQFISTDTVMTPPMMDDNVKDGSLLLRRPKTLTGNSTWRVRYGILRDDVLQLFDKNQLTETIKLRQSSIELIPNLPEDRFGTRNGFLITEHKKSGLSTSTKYYICTETSKERELWLSAFSDYIDPSQSLSLSSSRNANDTDSASHLSA	GHMKSDIPLFVQPEDFGTIQIEVLSTLYRDNEDDLSILIAIIDRKSGKEMFKFSKSIHKVRELDVYMKSHVPDLPLPTLPDRQLFQTLSPTKVDTRKNILNQYYTSIFSVPEFPKNVGLKIAQFISTDTVMTPPMMDDNVKDGSLLLRRPKTLTGNSTWRVRYGILRDDVLQLFDKNQLTETIKLRQSSIELIPNLPEDRFGTRNGFLITEHKKSGLSTSTKYYICTETSKERELWLSAFSDYIDPSQSLSLSS

**Table 2:** Crystallization conditions

Protein	Bem3_500–765_, SeMet K680M Bem3_500–750_, SeMet L688M Bem3_500–750_
Protein concentration (mg ml^−1^)	33–48
Temperature (K)	293
Method	Hanging-drop vapour diffusion
Drop ratio	1 µl + 1 µl
Buffer composition of protein solution	20 m*M* HEPES pH 8.0, 100 m*M* NaCl, 2 m*M* dithioerythritol, 5% glycerol
Reservoir solution	500 µl 0.1 *M* PCB pH 7.5–7.7, 17–19% PEG 1500

**Table 3:** Data-collection and processing statistics Values in parentheses are for the outer shell.

	Bem3_500–765_	SeMet K680M Bem3_500–750_ †	SeMet L688M Bem3_500–750_ †
Diffraction source	X10SA, SLS	X10SA, SLS	X10SA, SLS
Wavelength (Å)	0.98013	0.97794	0.97793
Temperature (K)	100	100	100
Detector	PILATUS 6M	PILATUS 6M	PILATUS 6M
Crystal-to-detector distance (mm)	400.0	400.0	450.0
Rotation range per image (°)	0.25	0.25	0.25
Total rotation range (°)	180	720	720
Exposure time per image (s)	0.1	0.1	0.1
Space group	*P*6_2_	*P*6_2_	*P*6_2_
*a*, *b*, *c* (Å)	85.37, 85.37, 63.97	85.80, 85.80, 65.01	85.57, 85.57, 64.02
α, β, γ (°)	90, 90, 120	90, 90, 120	90, 90, 120
Resolution range (Å)	42.7–2.2 (2.3–2.2)	48.9–2.9 (3.0–2.9)	48.4–2.6 (2.7–2.6)
Total No. of reflections	137033 (16361)	252326 (25224)	342485 (37132)
No. of unique reflections	13566 (1677)	11870 (1154)	16117 (1700)
Completeness (%)	100.0 (99.9)	100.0 (99.9)	100.0 (100.0)
*R*_merge_ (%)	4.5 (110.9)	8.0 (139.6)	9.9 (146.0)
*R*_meas_ (%)	4.7 (117.1)	8.2 (142.9)	10.2 (149.4)
Overall *B* factor from Wilson plot (Å^2^)	58	91	56
〈*I*/σ(*I*)〉	26.7 (2.2)	26.3 (2.1)	23.83 (2.1)
CC_1/2_	1.0 (0.747)	1.0 (0.895)	1.0 (0.753)

† Data statistics refer to unmerged Friedel pairs.

**Table 4:** Refinement statistics for Bem3_500–765_

Resolution range (Å)	42.7–2.2
Completeness (%)	99.96
No. of reflections	13565
No. of reflections, test set	679
Final *R*_cryst_ (%)	21.2
Final *R*_free_ (%)	23.4
R.m.s. deviations	
Bond lengths (Å)	0.002
Bond angles (°)	0.572
No. of non-H atoms	
Protein	1787
Water	21
Average *B* factors (Å^2^)	
Protein	81
Water	62
Ramachandran plot	
Favoured (%)	97.6
Allowed (%)	2.4
Outliers (%)	0
